# A Rare Cause of Pericardial Effusion: Giant Cell Arteritis

**DOI:** 10.1155/2014/424295

**Published:** 2014-01-02

**Authors:** Turker Tasliyurt, Hakan Sivgin, Lutfu Bekar, Safak Sahin, Suheyla Uzun Kaya, Resit Dogan Koseoglu, Faruk Kutluturk, Abdulkerim Yilmaz

**Affiliations:** ^1^Department of Internal Medicine, School of Medicine, Gaziosmanpasa University, Ali Sevki Erek Campus, 60100 Tokat, Turkey; ^2^Department of Cardiology, School of Medicine, Gaziosmanpasa University, 60100 Tokat, Turkey; ^3^Department of Pathology, School of Medicine, Gaziosmanpasa University, 60100 Tokat, Turkey

## Abstract

Giant cell arteritis is a granulomatous vasculitis characterized by medium or large sized vessel involvement. Although extracranial branches of the carotid artery are typically involved, involvement of aorta and its major branches can also be seen. Cardiac involvement has been encountered less frequently and pericardial effusion is rarely encountered. In this paper, a case has been presented in which pericardial effusion was determined during the examination and diagnosis was giant cell arteritis.

## 1. Introduction

Giant cell arteritis (GCA), also called temporal arteritis, is granulomatous vasculitis of major vessel. It is generally observed after 50 years of age and ratio of female/male patients is 2–4/1. It involves especially extracranial branches of carotid artery [1]. Based on this involvement, newly started headache, claudication in jaw and tongue, visual impairment symptoms, and temporal artery sensitivity develop. In addition, nonspecific systemic symptoms such as fever, weight and appetite loss, and fatigue may also appear as the initial symptoms of the disease. In about half of the patients, symptoms of polymyalgia rheumatica accompany the clinical manifestation [2].

Cardiac involvement is rare in GCA. Myocardial infarction and aortic aneurysm are serious manifestations that may arise [3–5]. Pericardial involvement in GCA is quite rare. In the present case, we report a GCA with pericardial effusion who had nonspecific symptoms such as weight loss and fatigue at first presentation.

## 2. Case Presentation

A 74-year-old female patient applied to our clinic with the complaints of fatigue, loss of appetite, weight loss. She had lost five kg in the last two months during which she had the complaints. In tests conducted by another center, high erythrocyte sedimentation rate (ESR) and C-reactive protein (CRP) values had been detected, and for evaluation she was referred to our hospital with malignity and infection prediagnoses. When detailed anamnesis was taken, she was found to have complaints of fever, one-sided headache, and claudication. She had no diseases other than hypertension from which she had suffered for 10 years.

Physical examination determined the following findings: body temperature, 36.8°C; heart rate, 80 beats/min and rhythmic; blood pressure, 130/80 mmHg. Left temporal artery was tender with palpation and scalp was aching. There was no pathological finding in lung and cardiac examination. In abdominal examination, there was no abnormality other than slight tenderness in right, upper quadrant upon deep palpation. Other system examinations were normal.

Laboratory test findings were as follows: ESR: 120 mm/hr, CRP: 181 mg/L (0–5), leucocyte: 10400/mm^3^ (4000–11000), Hgb: 9.9 g/dL (11–18), Plt: 611000/mm^3^ (150000–400000), MCV: 83 fL (80–100), ferritin: 473.6 ng/mL (13–150), ALT: 67 U/L (7–35), AST: 51 U/L (10–38), ALP: 307 U/L (35–270), GGT: 406 U/L (5–61), total bilirubin: 0.32 mg/dL (0.01–1.2), albumin: 2.3 gr/dL (3.4–5.5), INR: 1.23. Kidney and thyroid functions were within normal ranges and no abnormality was found in urine tests. Rheumatoid factor, anti-nuclear antibody, anti-neutrophilic cytoplasmic antibody, and brucellosis agglutination were negative. Immunoglobulin levels were normal. Hepatitis and other viral serological tests turned out to be negative. There was no characteristic in lung radiography and no pathology was detected in abdominal USG. The patient had no visual problems and fundoscopic examination did not reveal any abnormality.

The patient had high ESR and CRP values in addition to fever complaint. Procalcitonin value was 0.18 and no growth was observed in blood and urine cultures. No infection source was determined, and therefore antibiotic treatment was not started. In echocardiography of the patient, pericardial effusion was detected. Left ventricular diameter and functions were within normal limits. There was an effusion (10–12 mm) which surrounded the heart all the way but did not cause cardiac tamponade or hemodynamic instability. Electrocardiography was normal except for low voltage.

**Figure 1:**
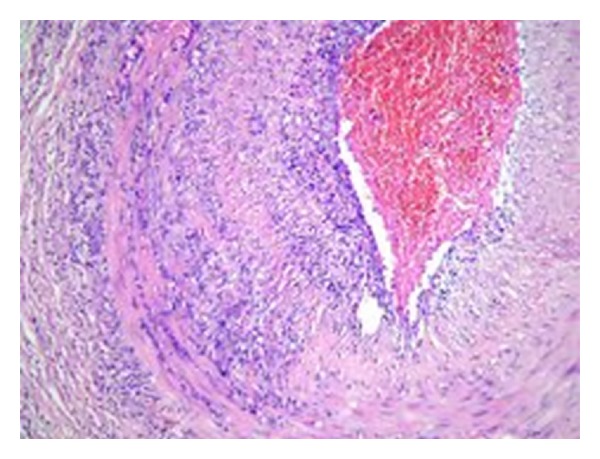
Mononuclear inflammatory cell infiltration in temporal artery from intima to the adventitia layer (HE, × 10).

**Figure 2:**
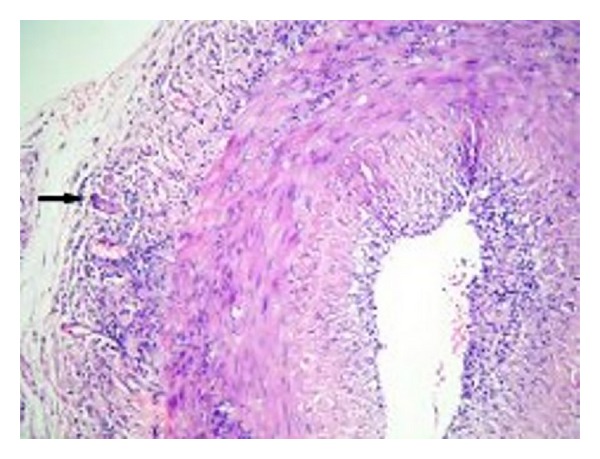
Multinuclear giant cells accompanying mononuclear inflammatory cell infiltration of artery wall in adventitia (arrow) (HE, × 10).

Based on the available clinical and laboratory findings, GCA was considered and temporal artery biopsy was conducted. Results of biopsy confirmed GCA (Figures 1 and 2). A 60 mg/day prednisolone treatment was started. After the treatment, clear improvements were observed in clinical conditions of the patient. A week later, ESR, CRP, and liver function tests decreased to normal levels. In the follow-up echocardiography, pericardial effusion was remarkably decreased. Steroid dose administered was gradually lowered. At the end of one year, the patient who was continuing to take 10 mg/day maintenance level of steroid was stable in terms of clinical status and laboratory values. In echocardiography, pericardial effusion was completely disappeared.

## 3. Discussion

GCA is a systemic vasculitis involving large and medium diameter arteries, predominantly aortic cranial branches. It is more common in women and after the age of 50. Although it has a comorbidity with polymyalgia rheumatica, it is less common. Besides the systemic symptoms such as fever, loss of appetite, fatigue, and weight loss, ischemic symptoms such as claudication in jaw and tongue, headache in temporal and occipital area, and sudden loss of vision which are specific to temporal arteritis can also be seen [1, 6]. In our case, no visual complaint existed, but other common symptoms of GCA were present.

Elevated ESR and CRP levels, impaired liver function tests, and anemia are frequently observed laboratory findings in GCA and were also evident in our case [6]. After the treatment, these abnormal laboratory values rapidly improved and came to normal limits.

GCA diagnosis is generally made based on the diagnosis criteria published by American College of Rheumatology in 1990. These criteria are age of over 50, newly started headache, ESR > 50 mm/hour, tenderness in temporal artery or decrease in pulsation, and granulomatous inflammation findings in temporal artery biopsy [7]. Presence of at least three criteria is enough for diagnosis. Sensitivity is 93.5% and specificity is 91.2%. In our case, temporal artery biopsy was in accordance with GCA, and other criteria were also observed.

Although extracranial branches of carotid artery are typically involved in GCA, involvement of aorta and major branches can be observed in 10–15% of the patients [8]. Aortic aneurysm can be observed as a late complication in 10% of the patients [4]. Narrowing and occlusion of carotis and vertebrobasilar arteries can be seen in 20–30% of the patients [9]. Cardiac involvement is less common and can be in the form of myocardial infarction, myocarditis, and aortic valve involvement. Pericardial effusion, on the other hand, is very rare [10, 11]. In our case, pericardial effusion was found after the tests. With steroid treatment after the diagnosis, clinical status and laboratory test results of the patient improved and pericardial effusion disappeared.

In conclusion, GCA patients may present themselves with nonspecific complaints such as fever, weight loss, and fatigue in addition to typical clinic signs of the disease. Delaying in diagnosis and in starting of the treatment has significant implications in terms of morbidity and mortality. It should be kept in mind that, although very rare, GCA can be accompanied with pericardial effusion as in our case.
